# Supplementary material for: Genome analysis and pleiotropy assessment using causal networks with loss of function mutation and metabolomics
Source: BMC Genomics. 2019 May 21;20:395. doi: 10.1186/s12864-019-5772-4 (PMC6528192; doi:10.1186/s12864-019-5772-4)
Supplement: Supplementary file 1 — Figure S1. European-American metabolomic causal network using the G-DAG algorithm. Table S1. Characteristics of ARIC Cohort. Metabolite assessment and Tables S2. and S3. for QC. Tables S4. and S5. LoF-metabolite relationships using the CCRS approach for EA and AA populations respectively. Tables S6. and S7. LoF-metabolite relationships using the single variant test for EA and AA populations respectively. Table S8. List of 122 named metabolites measured in ARIC study. (DOCX 974 kb) [file 12864_2019_5772_MOESM1_ESM.docx]

**Supplementary**

**Content**

1. Figure S1. European-American metabolomic causal network using the G-DAG algorithm.
2. Table S1. Characteristics of ARIC Cohort
3. Metabolite assessment and Tables S2 and S3 for QC.
4. Tables S4 and S5. LoF-metabolite relationships using the CCRS approach for EA and AA populations respectively.
5. Tables S6 and S7. LoF-metabolite relationships using the single variant test for EA and AA populations respectively.
6. Table S8. List of 122 named metabolites measured in ARIC study


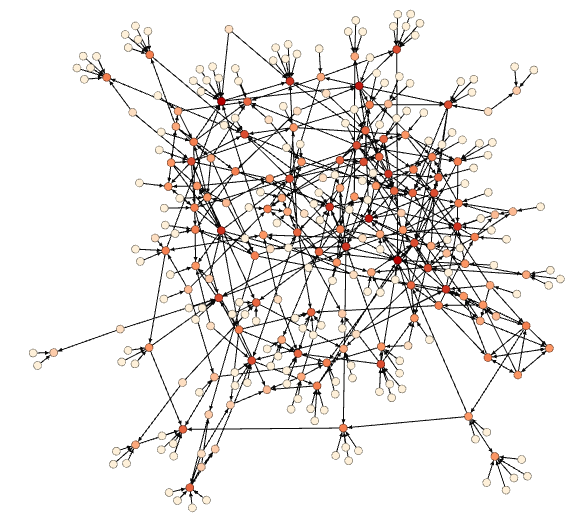

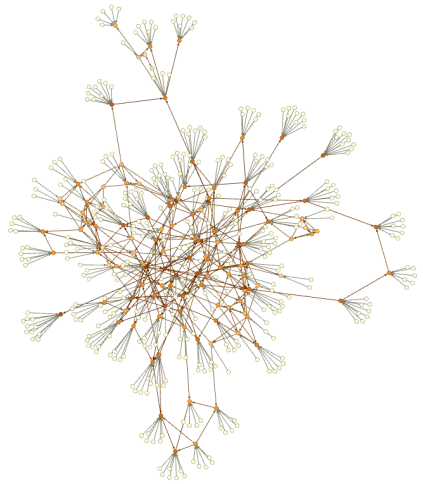


**Additional file 1: Figure S1**. European-American (left) and African-American (right) metabolomic causal network using the G-DAG algorithm. The white nodes are instrumental variables generated through extracting information from the genome. The other nodes stand for metabolites. For extrcating information from the G-DAG outcome, see the reference below.

Yazdani A, Yazdani A, Samiei A, Boerwinkle E. Identification, analysis, and interpretation of a human serum metabolomic causal network in an observational study. J Biomed Inform. 2016;63:337–43.

**Additional file 1: Table S1**. Characteristics of ARIC Cohort


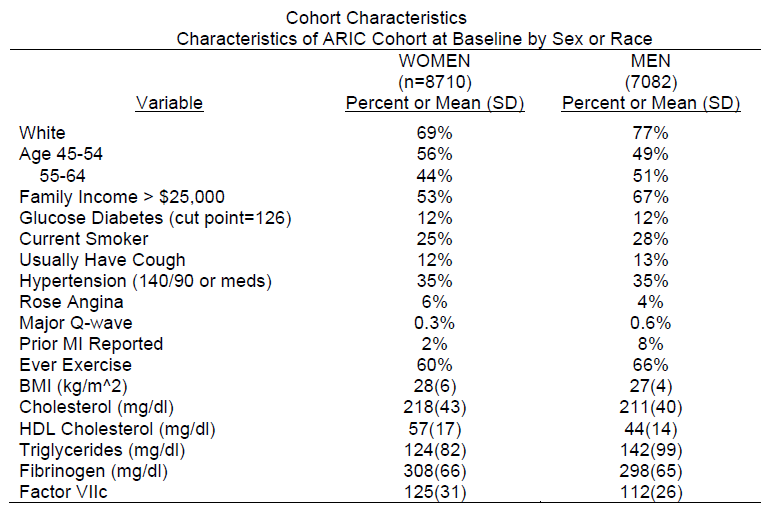


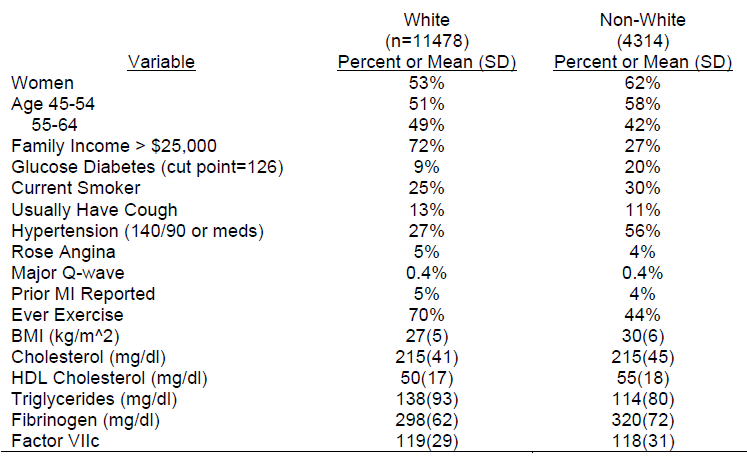


**3. Metabolite Assessment**

Metabolite profiling of fasting serum samples that had been stored at −80°C since collection at baseline in 1986–1987 was completed in June 2010. Detection and quantification of metabolites were completed by Metabolon, Inc. (Durham, North Carolina). Several types of internal controls were analyzed in concert with the experimental samples. Tables S2 and S3 describe these quality assurance and quality control samples and standards. Further QC consists of four major components: the LIMS, the data extraction and peak-identification software, data processing tools for QC and compound identification.


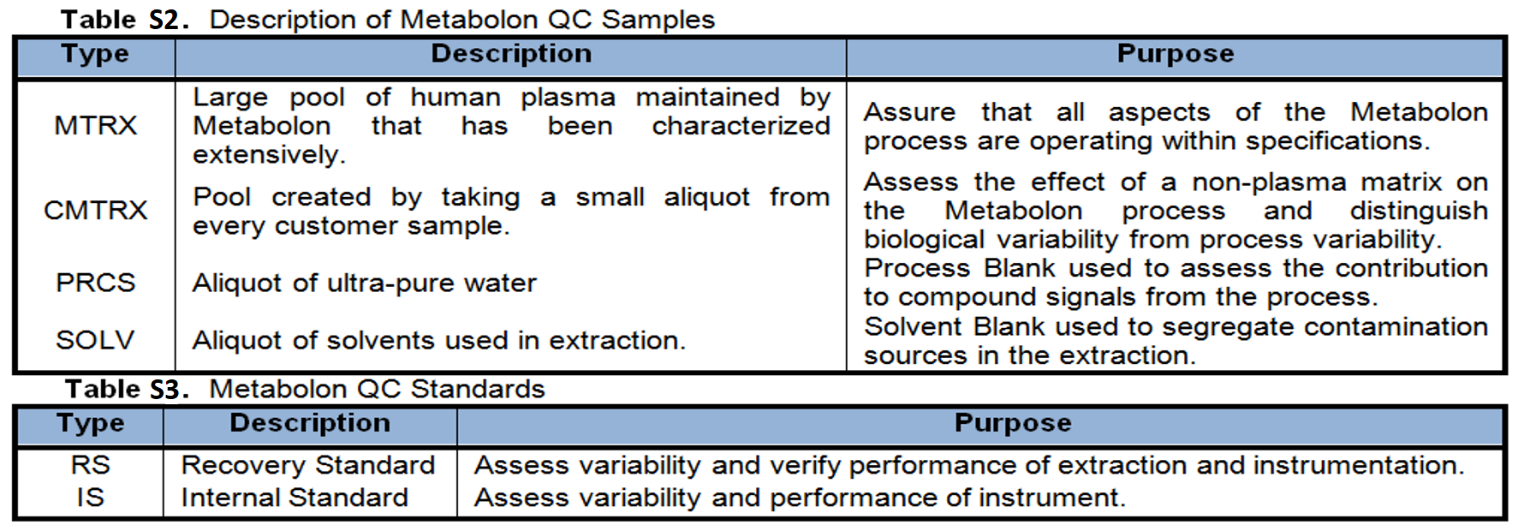


For detection and quantification of 602 metabolites an untargeted, gas chromatography–mass spectrometry and liquid chromatography–mass spectrometry–based metabolomic quantification protocol (1) were utilized. This untargeted approach identifies and quantifies named compounds whose chemical identities are known (n = 361), as well as additional unnamed compounds that do not currently have chemical standards (n = 241) (2). These unnamed compounds are tagged beginning with “X” and followed by numbers (e.g., X-12345). A rigorous assessment of the metabolomic data was done on the basis of 2 criteria. First, 44 metabolites were excluded because more than 50% of the samples had missing values or values below the detection limit of the technology. Considering the appropriateness of population-based referencing, we preferred a relatively stable biomarker with a small intrasubject variance compared with the between-subject variance (3). Second, 386 metabolites were excluded because the medium-term reliability coefficient based on a comparison of 2 amples collected 4–6 weeks apart from 60 individuals was less than 0.60. Of note, 114 metabolites were excluded because they had more than 50% missing values and reliability coefficients of less than 0.60. Thus, the present report is based on an evaluation of 122 stable metabolites. Instrument variability was determined by calculating the median relative standard deviation for the internal standards that were added to each sample prior to injection into the mass spectrometers. Overall process variability was determined by calculating the median relative standard deviation for all endogenous metabolites (i.e., non instrument standards) present in 100% of the technical replicate samples, which were created from a homogeneous pool of human plasma. Because this study spanned multiple days, a data normalization step was performed to correct variation resulting from differences in instrument tuning from day to day. Essentially, each compound was corrected in run-day blocks by registering the medians to equal 1.00 and normalizing each data point proportionately.

Compounds were identified by comparison with an in house–generated authentic standard library that includes retention times, molecular weights, preferred adducts, in-source fragments, and associated fragmentation spectra of the intact parent ions. The database allows for rapid and high-confidence identification of experimentally detected molecules on a multi parameter match basis without the need for additional analyses. Unnamed compounds of interest were subjected to detailed analysis by mass spectrometry and the accurate monoisotopic mass and fragmentation patterns of the primary ion, as well as higher order fragmentations by using an Or bitrap Elite instrument (Thermo Fisher Scientific, Inc., Waltham, Massachusetts). Resulting empirical formulas were queried against the Chem Spider database (<http://www.chem> spider.com/) search engine for potential structure matches. Most of the text above was published previously (4).

References

1. Yazdani A, Yazdani A, Samiei A, Boerwinkle E. Identification, analysis, and interpretation of a human serum metabolomics causal network in an observational study. J Biomed Inform. 2016;63:337–43.

**Additional file 1: Table S4.** EA (European-American/white) population, LoF-metabolite relationships using the CCRS approach. Note that CCRS is a penalizatin-based approach and simoultanously estimate coefficient effects and select the variants.

| **SNP** | **Metabolite** | **Effect Size** |
| --- | --- | --- |
| **X6.32134395.C.G** | myristoleate | 0.85 |
| **X19.58153465.T.A** | myristoleate | 0.6 |
| **X19.549171.C.T** | eicosenoate | 0.5 |
| **X6.150390149.G.C** | azelate | 0.5 |
| **X1.145074975.G.A** | dodecanedioate | 0.57 |
| **X19.549171.C.T** | octanoylcarnitine | 0.55 |
| **X19.549171.C.T** | decanoylcarnitine | 0.52 |
| **X5.179280392.G.A** | cis4decenoylcarnitine | 1.04 |
| **X6.116600895.G.A** | pregnsteroidmonosulfate | 0.61 |
| **X11.71807767.A.C** | betaine | 0.67 |
| **X17.15546132.T.TA** | kynurenine | 0.51 |
| **X21.10907041.C.A** | isovalerylcarnitine | 0.64 |
| **X21.10907041.C.A** | isobutyrylcarnitine | 0.76 |
| **X10.50901917.C.A** | hydroxybutyrate | 0.51 |
| **X21.42551432.T.A** | trans4hydroxyproline | 0.68 |
| **X7.72419008.G.C** | prohydroxypro | 0.54 |
| **X14.94754643.C.T** | prohydroxypro | 0.58 |
| **X2.138030234.C.T** | glycylvaline | 0.69 |
| **X7.23313823.G.T** | lactate | 0.55 |
| **X1.228469903.A.T** | mannose | 0.55 |
| **X16.726189.C.T** | phosphate | 0.73 |
| **X12.57441459.G.A** | heptanoate | 0.52 |
| **X19.22575784.C.A** | docosahexaenoate | 0.56 |
| **X10.90354423.G.C** | dihomolinolenate | 0.52 |
| **X17.15546132.T.TA** | dihomolinolenate | 0.56 |
| **X11.71807767.A.C** | carboxy4methyl5propyl2furanpropanoate | 0.61 |
| **X12.52799923.G.A** | propionylcarnitine | 0.5 |
| **X21.10907041.C.A** | propionylcarnitine | 0.63 |
| **X16.67220231.G.A** | HETE | 0.52 |
| **X19.3543380.C.G** | HETE | 0.5 |
| **X11.71807767.A.C** | myoinositol | 0.5 |
| **X21.10907041.C.A** | myoinositol | 0.67 |
| **X16.1484536.C.T** | glycerophosphorylcholine | 0.52 |
| **X12.57441459.G.A** | glycerol | 0.75 |
| **X11.62996038.T.C** | pregnendioldisulfate | 0.57 |
| **X21.42551432.T.A** | androsten3beta17betadioldisulfate1 | 0.71 |
| **X15.41796352.C.A** | glycocholenatesulfate | 0.81 |
| **X21.10907041.C.A** | OsulfoLtyrosine | 0.68 |

**Additional file 1: Table S5.** AA (African-American/non-white) population, LoF-metabolite relationships using the CCRS approach. Note that CCRS is a penalizatin-based approach and simoultanously estimate coefficient effects and select the variants.

| **SNP** | **Metabolite** | **Effect**  **Size** |
| --- | --- | --- |
| **X2.207041527.G.A** | glutarate | 0.53 |
| **X10.1569095.G.A** | glutarate | 0.56 |
| **X1.156563265.C.T** | leucine | 1.09 |
| **X6.31692558.C.T** | leucine | 0.56 |
| **X16.48204130.C.T** | leucine | 0.65 |
| **X19.51411852.C.T** | leucine | 0.5 |
| **X6.31692558.C.T** | isoleucine | 0.58 |
| **X21.31538461.G.A** | isoleucine | 0.74 |
| **X.57934556.C.A** | gammaglutamylalanine | 0.54 |
| **X1.24727815.G.T** | gammaglutamylglutamate | 1.08 |
| **X15.93162691.T.C** | gammaglutamylglutamate | 0.5 |
| **X17.40717487.A.G** | gammaglutamylglutamate | 0.65 |
| **X19.41351363.T.A** | gammaglutamylisoleucine | 0.89 |
| **X19.41351363.T.A** | gammaglutamylleucine | 1.41 |
| **X19.41351363.T.A** | gammaglutamylphenylalanine | 0.69 |
| **X19.41351363.T.A** | gammaglutamylthreonine | 1.28 |
| **X19.41351363.T.A** | gammaglutamylvaline | 0.91 |
| **X2.207041527.G.A** | glycylleucine | 0.53 |
| **X8.101629847.C.T** | glycylleucine | 0.53 |
| **X8.101629847.C.T** | leucylalanine | 0.63 |
| **X15.93162691.T.C** | leucylalanine | 0.5 |
| **X19.40554705.T.C** | margarate170 | 0.56 |
| **X21.33954602.G.A** | margarate170 | 0.59 |
| **X1.232942469.G.A** | oleate181n9 | 0.88 |
| **X16.57707232.G.C** | oleate181n9 | 0.72 |
| **X1.232942469.G.A** | eicosenoate201n9 | 0.89 |
| **X16.57707232.G.C** | eicosenoate201n9 | 0.82 |
| **X2.207041527.G.A** | adipate | 0.64 |
| **X17.40342211.G.A** | adipate | 0.84 |
| **X20.44511257.G.A** | dodecanedioate | 0.9 |
| **X1.151016171.G.A** | octanoylcarnitine | 0.97 |
| **X17.39888946.C.A** | octanoylcarnitine | 0.88 |
| **X1.151016171.G.A** | decanoylcarnitine | 0.95 |
| **X2.207041527.G.A** | decanoylcarnitine | 0.52 |
| **X17.39888946.C.A** | decanoylcarnitine | 0.92 |
| **X1.230927675.G.A** | cis4decenoylcarnitine | 0.58 |
| **X1.157504478.G.T** | laurylcarnitine | 0.54 |
| **X3.178962425.C.T** | arachidonoylglycerophosphocholine204n6 | 0.52 |
| **X5.56778417.G.A** | docosapentaenoylglycerophosphocholine225n31 | 0.50 |
| **X6.31743860.G.A** | docosapentaenoylglycerophosphocholine225n31 | 0.53 |
| **X6.31743860.G.A** | docosahexaenoylglycerophosphocholine226n3 | 0.58 |
| **X17.72540958.G.A** | docosahexaenoylglycerophosphoethanolamine1 | 0.96 |
| **X15.85438665.G.A** | androsten3beta17betadioldisulfate2 | 0.56 |
| **X16.89724661.G.T** | androsten3beta17betadioldisulfate2 | 0.81 |
| **X2.207041527.G.A** | glycine | 0.51 |
| **X19.58213743.G.A** | glycine | 0.52 |
| **X1.157504478.G.T** | betaine | 0.59 |
| **X1.36208741.C.T** | serine | 0.55 |
| **X19.41594954.C.T** | pyroglutamine | 0.7 |
| **X2.55407644.A.T** | lysine | 0.54 |
| **X3.108672558.C.A** | glutarylcarnitine | 0.98 |
| **X6.31692558.C.T** | glutarylcarnitine | 0.5 |
| **X19.51411852.C.T** | glutarylcarnitine | 0.50 |
| **X1.151016171.G.A** | phenylalanine | 0.5 |
| **X17.40342211.G.A** | phenylalanine | 0.57 |
| **X3.36756821.A.C** | phenylacetylglutamine | 0.71 |
| **X19.42930751.G.T** | phenylacetylglutamine | 0.52 |
| **X20.44511257.G.A** | phenylacetylglutamine | 1.28 |
| **X21.43792873.A.G** | phenylacetylglutamine | 0.89 |
| **X2.207041527.G.A** | tyrosine | 0.66 |
| **X19.53014055.C.T** | tyrosine | 0.56 |
| **X10.1569095.G.A** | hydroxyphenyllactate | 0.5 |
| **X19.41594954.C.T** | hydroxyphenyllactate | 0.9 |
| **X12.7899913.C.A** | isovalerate | 0.58 |
| **X21.42818050.G.T** | isovalerate | 0.5 |
| **X4.69095086.C.A** | isobutyrylcarnitine | 0.52 |
| **X19.49376719.A.T** | aminobutyrate | 0.52 |
| **X2.130931095.G.A** | hydroxybutyrate2 | 0.56 |
| **X1.36208741.C.T** | urea | 1 |
| **X15.34159987.G.A** | urea | 0.7 |
| **X15.41799325.G.A** | urea | 0.51 |
| **X22.25599863.G.T** | trans4hydroxyproline | 0.66 |
| **X16.48250144.C.A** | prohydroxypro | 0.54 |
| **X1.24727815.G.T** | oxoproline | 0.9 |
| **X3.38348743.A.C** | gammaglutamyltyrosine | 0.85 |
| **X12.7899913.C.A** | glycylvaline | 0.55 |
| **X1.232942469.G.A** | mannose | 0.5 |
| **X19.42132032.G.A** | succinate | 0.77 |
| **X1.230927675.G.A** | heptanoate70 | 0.7 |
| **X2.207041527.G.A** | heptanoate70 | 0.57 |
| **X12.7899913.C.A** | nonadecanoate190 | 0.99 |
| **X1.24727815.G.T** | eicosapentaenoate205n3 | 0.69 |
| **X17.65989037.A.G** | eicosapentaenoate205n3 | 0.54 |
| **X20.36869005.G.A** | eicosapentaenoate205n3 | 0.85 |
| **X12.51639869.C.T** | arachidonate204n6 | 0.51 |
| **X17.65989037.A.G** | arachidonate204n6 | 0.56 |
| **X2.207041527.G.A** | glycerophosphorylcholine | 0.61 |
| **X6.56044484.C.A** | glycerophosphorylcholine | 0.59 |
| **X22.25599863.G.T** | oleoylglycerophosphocholine181 | 0.57 |
| **X3.56628033.C.T** | palmitoylplasmenylethanolamine | 0.57 |
| **X6.105606599.G.A** | palmitoylplasmenylethanolamine | 0.54 |
| **X15.93162691.T.C** | arachidonoylglycerophosphoinositol | 0.54 |
| **X11.118827917.G.A** | glycerol | 0.79 |
| **X21.31538461.G.A** | glycerol | 0.67 |
| **X15.93015466.A.T** | glycerol3phosphate | 0.51 |
| **X21.33954602.G.A** | glycerol3phosphate | 0.57 |
| **X3.186389555.C.T** | cholesterol | 0.55 |
| **X9.73152037.C.T** | cholesterol | 0.76 |
| **X1.55075006.G.A** | pregnendioldisulfate | 1.16 |
| **X16.57707232.G.C** | androsten3beta17betadioldisulfate1 | 0.5 |
| **X19.38875072.G.A** | androsten3beta17betadioldisulfate1 | 0.69 |
| **X22.25599863.G.T** | androsten3beta17betadioldisulfate1 | 0.51 |
| **X16.57707232.G.C** | glycocholenatesulfate | 1.09 |
| **X19.38875072.G.A** | xanthine | 0.62 |
| **X19.56482063.G.A** | xanthine | 0.53 |
| **X2.207041527.G.A** | pseudouridine | 0.54 |
| **X12.7899913.C.A** | pseudouridine | 0.54 |
| **X17.40342211.G.A** | N1Methyl2pyridone5carboxamide | 0.53 |
| **X19.42132032.G.A** | N1Methyl2pyridone5carboxamide | 0.55 |
| **X.100274272.C.T** | N1Methyl2pyridone5carboxamide | 0.6 |
| **X19.35718891.C.T** | hippurate | 0.52 |
| **X.74334588.C.T** | catecholsulfate | 0.58 |
| **X19.48523114.G.A** | erythritol | 1.31 |
| **X6.46623768.G.A** | OsulfoLtyrosine | 0.61 |
| **X17.39888946.C.A** | OsulfoLtyrosine | 0.78 |

**Additional file 1: Table S6**. EA (European-American/white) population, LoF-metabolite relationships using the single variant test at level 4e-07 or smaller.

| SNP | Metabolite | P-value |
| --- | --- | --- |
| X15.50593417.C.A | isoleucine | 1.36E-07 |
| X6.32134395.C.G | myristoleate141n5 | 1.25E-12 |
| X19.58153465.T.A | myristoleate141n5 | 6.16E-10 |
| X19.22846601.G.T | heptadecenoate171n7 | 2.18E-07 |
| X19.549171.C.T | eicosenoate201n9 | 7.58E-07 |
| X6.150390149.G.C | azelate | 2.16E-07 |
| X1.145074975.G.A | dodecanedioate | 6.03E-15 |
| X19.549171.C.T | octanoylcarnitine | 3.77E-08 |
| X19.549171.C.T | decanoylcarnitine | 3.59E-08 |
| X2.120439282.C.T | cis4decenoylcarnitine | 3.01E-08 |
| X5.179280392.G.A | cis4decenoylcarnitine | 3.17E-19 |
| X12.11174286.C.T | cis4decenoylcarnitine | 5.86E-07 |
| X22.20024596.C.G | cis4decenoylcarnitine | 2.76E-07 |
| X6.116600895.G.A | pregnsteroidmonosulfate | 1.46E-07 |
| X11.71807767.A.C | betaine | 6.13E-09 |
| X1.228469903.A.T | serine | 5.80E-07 |
| X2.178879181.G.A | serine | 1.32E-07 |
| X10.50901917.C.A | hydroxybutyrate2 | 3.27E-07 |
| X3.130187662.G.T | urea | 4.71E-07 |
| X4.76521525.C.T | trans4hydroxyproline | 6.36E-07 |
| X14.94754643.C.T | prohydroxypro | 1.88E-08 |
| X2.138030234.C.T | glycylvaline | 1.99E-12 |
| X7.23313823.G.T | lactate | 2.87E-10 |
| X1.228469903.A.T | mannose | 1.20E-07 |
| X14.68053899.C.T | citrate | 5.67E-08 |
| X16.726189.C.T | phosphate | 2.27E-11 |
| X12.57441459.G.A | heptanoate70 | 2.55E-09 |
| X12.52711747.C.A | adrenate224n6 | 8.53E-08 |
| X1.24447867.C.T | eicosanodioate | 2.52E-09 |
| X11.71807767.A.C | myoinositol | 9.22E-09 |
| X11.62996038.T.C | myoinositol | 2.45E-08 |
| X21.10907041.C.A | myoinositol | 1.51E-10 |
| X16.1484536.C.T | glycerophosphorylcholine | 1.09E-07 |
| X12.11174286.C.T | oleoylglycerophosphocholine181 | 1.40E-08 |
| X12.57441459.G.A | glycerol | 3.54E-07 |
| X17.38519831.G.T | cholesterol | 2.98E-09 |
| X21.42551432.T.A | androsten3beta17betadioldisulfate1 | 3.77E-09 |
| X15.41796352.C.A | glycocholenatesulfate | 3.70E-09 |
| X1.41486083.C.A | pseudouridine | 1.70E-08 |
| X21.39528455.A.T | hippurate | 9.48E-08 |
| X21.10907041.C.A | OsulfoLtyrosine | 5.57E-13 |
| X1.26644537.C.A | OsulfoLtyrosine | 1.06E-07 |
| X12.25705804.C.T | OsulfoLtyrosine | 3.06E-08 |
| X19.42092225.G.A | OsulfoLtyrosine | 2.91E-09 |

**Additional file 1: Table S7**. AA (African-American/non-white) population LoF-metabolite relationships using the single variant test at statistical level 1e-6 or smaller.

| SNP | Metabolite | P-value |
| --- | --- | --- |
| X17.6493199.G.A | leucine | 4.41E-07 |
| X1.156563265.C.T | leucine | 3.74E-13 |
| X3.132378559.C.A | leucine | 6.22E-07 |
| X6.31692558.C.T | leucine | 3.61E-07 |
| X16.48204130.C.T | leucine | 3.16E-07 |
| X6.31692558.C.T | isoleucine | 3.67E-09 |
| X3.48501819.G.A | isoleucine | 1.53E-07 |
| X21.31538461.G.A | isoleucine | 7.15E-12 |
| X6.31692558.C.T | valine | 6.48E-07 |
| X.57934556.C.A | gammaglutamylalanine | 8.23E-07 |
| X1.24727815.G.T | gammaglutamylglutamate | 9.42E-13 |
| X17.40717487.A.G | gammaglutamylglutamate | 4.42E-09 |
| X14.20666175.C.A | gammaglutamylisoleucine | 3.47E-10 |
| X19.41351363.T.A | gammaglutamylisoleucine | 1.14E-10 |
| X14.20666175.C.A | gammaglutamylleucine | 9.88E-10 |
| X19.41351363.T.A | gammaglutamylleucine | 3.01E-20 |
| X14.20666175.C.A | gammaglutamylphenylalanine | 1.38E-08 |
| X14.20666175.C.A | gammaglutamylthreonine | 3.00E-10 |
| X19.41351363.T.A | gammaglutamylthreonine | 2.18E-17 |
| X14.20666175.C.A | gammaglutamylvaline | 4.72E-11 |
| X19.41351363.T.A | gammaglutamylvaline | 3.60E-11 |
| X.100274272.C.T | gammaglutamylvaline | 6.59E-07 |
| X2.207041527.G.A | glycylleucine | 4.15E-08 |
| X8.101629847.C.T | glycylleucine | 1.67E-07 |
| X8.101629847.C.T | leucylalanine | 2.01E-08 |
| X1.232942469.G.A | oleate181n9 | 6.20E-09 |
| X16.57707232.G.C | oleate181n10 | 5.30E-07 |
| X1.232942469.G.A | eicosenoate201n9 | 3.22E-08 |
| X16.57707232.G.C | eicosenoate201n10 | 1.91E-07 |
| X9.21166236.C.A | linolenate183n3 | 6.94E-09 |
| X2.207041527.G.A | adipate | 3.36E-07 |
| X20.44511257.G.A | dodecanedioate | 1.28E-07 |
| X1.151016171.G.A | octanoylcarnitine | 2.65E-10 |
| X17.39888946.C.A | octanoylcarnitine | 1.24E-09 |
| X1.151016171.G.A | decanoylcarnitine | 7.19E-10 |
| X17.39888946.C.A | decanoylcarnitine | 7.62E-10 |
| X3.178962425.C.T | arachidonoylglycerophosphocholine204n6 | 1.05E-08 |
| X17.8274704.C.T | arachidonoylglycerophosphocholine204n7 | 8.44E-08 |
| X17.72540958.G.A | docosahexaenoylglycerophosphoethanolamine1 | 2.27E-12 |
| X15.85438665.G.A | androsten3beta17betadioldisulfate2 | 3.71E-08 |
| X16.89724661.G.T | androsten3beta17betadioldisulfate3 | 4.11E-16 |
| X12.96380932.G.A | betaine | 1.10E-06 |
| X19.41594954.C.T | pyroglutamine | 3.27E-07 |
| X3.108672558.C.A | glutarylcarnitine | 2.02E-11 |
| X20.44511257.G.A | phenylacetylglutamine | 3.31E-15 |
| X2.108863758.G.A | phenylacetylglutamine | 4.56E-07 |
| X3.36756821.A.C | phenylacetylglutamine | 3.16E-09 |
| X21.43792873.A.G | phenylacetylglutamine | 1.79E-16 |
| X2.207041527.G.A | tyrosine | 9.14E-10 |
| X19.41594954.C.T | hydroxyphenyllactate | 1.13E-08 |
| X2.135988236.G.A | isovalerylcarnitine | 8.88E-07 |
| X19.57646393.T.A | isovalerylcarnitine | 1.15E-07 |
| X19.53668419.G.A | hydroxybutyrate2 | 3.46E-07 |
| X1.36208741.C.T | urea | 1.58E-13 |
| X15.34159987.G.A | urea | 7.14E-09 |
| X15.41799325.G.A | urea | 4.53E-09 |
| X22.25599863.G.T | trans4hydroxyproline | 3.34E-09 |
| X.154290120.A.G | trans4hydroxyproline | 1.44E-09 |
| X1.24727815.G.T | oxoproline | 3.98E-08 |
| X3.38348743.A.C | gammaglutamyltyrosine | 1.59E-07 |
| X19.42132032.G.A | succinate | 7.62E-08 |
| X3.49842325.G.T | phosphate | 3.99E-11 |
| X1.230927675.G.A | heptanoate70 | 1.69E-07 |
| X11.124761561.C.T | nonadecanoate190 | 9.66E-07 |
| X12.7899913.C.A | nonadecanoate191 | 3.53E-10 |
| X1.24727815.G.T | eicosapentaenoate205n3 | 3.38E-07 |
| X20.36869005.G.A | eicosapentaenoate205n3 | 3.72E-14 |
| X2.24302464.C.T | eicosanodioate | 1.27E-07 |
| X16.21279025.C.A | carboxy4methyl5propyl2furanpropanoate | 1.01E-07 |
| X19.51411852.C.T | carboxy4methyl5propyl2furanpropanoate | 6.82E-07 |
| X4.110757188.C.A | propionylcarnitine | 1.34E-08 |
| X3.38348743.A.C | HETE | 6.72E-07 |
| X6.56044484.C.A | glycerophosphorylcholine | 4.75E-08 |
| X6.56044484.C.A | glycerophosphorylcholine | 2.36E-07 |
| X8.143922642.G.A | palmitoylglycerophosphoinositol | 2.56E-08 |
| X21.31538461.G.A | glycerol | 9.69E-10 |
| X11.118827917.G.A | glycerol | 2.39E-10 |
| X9.37495945.C.A | glycerol3phosphate | 7.25E-08 |
| X15.93015466.A.T | glycerol3phosphate | 3.04E-09 |
| X9.73152037.C.T | cholesterol | 4.24E-07 |
| X1.55075006.G.A | pregnendioldisulfate | 5.80E-14 |
| X19.38875072.G.A | androsten3beta17betadioldisulfate1 | 1.06E-09 |
| X19.47192955.T.C | androsten3beta17betadioldisulfate1 | 3.48E-07 |
| X16.57707232.G.C | glycocholenatesulfate | 1.79E-11 |
| X19.38875072.G.A | xanthine | 2.98E-09 |
| X1.152681560.C.A | pseudouridine | 3.04E-08 |
| X.100274272.C.T | N1Methyl2pyridone5carboxamide | 2.34E-07 |
| X.74334588.C.T | catecholsulfate | 1.77E-08 |
| X19.48523114.G.A | erythritol | 1.64E-11 |
| X6.46623768.G.A | OsulfoLtyrosine | 7.27E-10 |

**Additional file 1: Table S8**. The super‐pathway, sub-pathway, and measurement platform for 122 metabolites measured in African-Americans population in ARIC study.

| BIOCHEMICAL | SUPER_  PATHWAY | SUB_  PATHWAY | PLATFORM |
| --- | --- | --- | --- |
| Glutarate | Amino acid | Lysine metabolism | GC/MS |
| p-cresol sulfate | Amino acid | Phenylalanine & tyrosine metabolism | LC/MS neg |
| Leucine | Amino acid | Valine, leucine and isoleucine metabolism | LC/MS pos |
| Isoleucine | Amino acid | Valine, leucine and isoleucine metabolism | LC/MS pos |
| Valine | Amino acid | Valine, leucine and isoleucine metabolism | LC/MS pos |
| gamma-glutamylalanine | Peptide | gamma-glutamyl | LC/MS pos |
| gamma-glutamylglutamate | Peptide | gamma-glutamyl | LC/MS pos |
| gamma-glutamylisoleucine | Peptide | gamma-glutamyl | LC/MS pos |
| gamma-glutamylleucine | Peptide | gamma-glutamyl | LC/MS pos |
| gamma-glutamylphenylalanine | Peptide | gamma-glutamyl | LC/MS pos |
| gamma-glutamylthreonine | Peptide | gamma-glutamyl | LC/MS pos |
| gamma-glutamylvaline | Peptide | gamma-glutamyl | LC/MS pos |
| Glycylleucine | Peptide | Dipeptide | LC/MS pos |
| Leucylalanine | Peptide | Dipeptide | LC/MS pos |
| Serylleucine | Peptide | Dipeptide |  |
| Caproate | Lipid | Medium chain fatty acid | LC/MS neg |
| Myristate | Lipid | Long chain fatty acid | LC/MS neg |
| Myristoleate | Lipid | Long chain fatty acid | LC/MS neg |
| Palmitate | Lipid | Long chain fatty acid | LC/MS neg |
| Palmitoleate | Lipid | Long chain fatty acid | LC/MS neg |
| Margarate | Lipid | Long chain fatty acid | LC/MS neg |
| heptadecanoate | Lipid | Long chain fatty acid | LC/MS neg |
| stearate180 | Lipid | Long chain fatty acid | LC/MS neg |
| oleate181n9 | Lipid | Long chain fatty acid | GC/MS |
| Nonadecenoate | Lipid | Long chain fatty acid | LC/MS neg |
| Eicosenoate | Lipid | Long chain fatty acid | LC/MS neg |
| Linoleate | Lipid | Long chain fatty acid | LC/MS neg |
| Linolenate | Lipid | Long chain fatty acid | LC/MS neg |
| dihomo-linoleate | Lipid | Long chain fatty acid | LC/MS neg |
| Adipate | Lipid | Fatty acid, dicarboxylate | GC/MS |
| Azelate | Lipid | Fatty acid, dicarboxylate | LC/MS neg |
| sebacate | Lipid | Fatty acid, dicarboxylate | LC/MS neg |
| dodecanedioate | Lipid | Fatty acid, dicarboxylate | LC/MS neg |
| octanoylcarnitine | Lipid | Carnitine metabolism | LC/MS pos |
| decanoylcarnitine | Lipid | Carnitine metabolism | LC/MS pos |
| cis4decenoylcarnitine | Lipid | Carnitine metabolism | LC/MS pos |
| laurylcarnitine | Lipid | Carnitine metabolism | LC/MS pos |
| hydroxypalmitate2 | Lipid | Fatty acid, monohydroxy | LC/MS neg |
| hydroxystearate | Lipid | Fatty acid, monohydroxy | LC/MS neg |
| 9-HODE | Lipid | Fatty acid, monohydroxy | LC/MS neg |
| Choline | Lipid | Glycerolipid metabolism | LC/MS pos |
| Arachidonoylglycerophosphocholine | Lipid | Lysolipid | LC/MS neg |
| docosapentaenoyl glycerophosphocholine | Lipid | Lysolipid | LC/MS pos |
| docosahexaenoylglycerophosphocholine | Lipid | Lysolipid | LC/MS pos |
| hydroxypregnenolonedisulfate | Lipid | Sterol/Steroid | LC/MS neg |
| pregnsteroidmonosulfate | Lipid | Sterol/Steroid | LC/MS neg |
| androsten3beta17betadioldisulfate2 | Lipid | Sterol/Steroid | LC/MS neg |
| erythritol | Xenobiotics | Sugar, sugar substitute, starch | GC/MS |
| Glycine | Amino acid | Glycine, serine and threonine metabolism | GC/MS |
| Betaine | Amino acid | Glycine, serine and threonine metabolism | LC/MS pos |
| Serine | Amino acid | Glycine, serine and threonine metabolism | GC/MS |
| threonine | Amino acid | Glycine, serine and threonine metabolism | LC/MS pos |
| Alanine | Amino acid | Alanine and aspartate metabolism | GC/MS |
| N-acetylalanine | Amino acid | Alanine and aspartate metabolism | LC/MS neg |
| glutamate | Amino acid | Glutamate metabolism | LC/MS neg |
| pyroglutamine | Amino acid | Glutamate metabolism | LC/MS pos |
| Lysine | Amino acid | Lysine metabolism | LC/MS pos |
| glutaryl carnitine | Amino acid | Lysine metabolism | LC/MS pos |
| phenylalanine | Amino acid | Phenylalanine & tyrosine metabolism | LC/MS pos |
| phenylacetylglutamine | Amino acid | Phenylalanine & tyrosine metabolism | LC/MS pos |
| tyrosine | Amino acid | Phenylalanine & tyrosine metabolism | LC/MS pos |
| hydroxyphenyllactate | Amino acid | Phenylalanine & tyrosine metabolism | GC/MS |
| tryptophan | Amino acid | Tryptophan metabolism | LC/MS pos |
| kynurenine | Amino acid | Tryptophan metabolism | LC/MS pos |
| isovalerate | Lipid | Fatty acid metabolism | LC/MS neg |
| isovalerylcarnitine | Amino acid | Valine, leucine and isoleucine metabolism | LC/MS pos |
| isobutyrylcarnitine | Amino acid | Valine, leucine and isoleucine metabolism | LC/MS pos |
| methioninesulfoxide | Amino acid | Cysteine, methionine, SAM, taurine metabolism | LC/MS pos |
| aminobutyrate | Amino acid | Butanoate metabolism | LC/MS pos |
| hydroxybutyrate2 | Amino acid | Cysteine, methionine, SAM, taurine metabolism | GC/MS |
| Urea | Amino acid | Urea cycle; arginine-, proline-, metabolism | GC/MS |
| Proline | Amino acid | Urea cycle; arginine-, proline-, metabolism | LC/MS pos |
| trans-4-hydroxyproline | Amino acid | Urea cycle; arginine-, proline-, metabolism | LC/MS pos |
| guanidinobutanoate | Amino acid | Guanidino and acetamido metabolism | LC/MS pos |
| oxoproline | Amino acid | Glutathione metabolism | LC/MS neg |
| gammaglutamyltyrosine | Peptide | gamma-glutamyl | LC/MS pos |
| glycylvaline | Peptide | Dipeptide | LC/MS pos |
| Lactate | Carbohydrate | Glycolysis, gluconeogenesis, pyruvate metabolism | GC/MS |
| mannose | Carbohydrate | Fructose, mannose, galactose, starch, and sucrose metabolism | GC/MS |
| Citrate | Energy | Krebs cycle | GC/MS |
| succinate | Energy | Krebs cycle | LC/MS neg |
| phosphate | Energy | Oxidative phosphorylation | GC/MS |
| heptanoate | Lipid | Medium chain fatty acid | LC/MS neg |
| Laurate | Lipid | Medium chain fatty acid | LC/MS neg |
| nonadecanoate | Lipid | Long chain fatty acid | LC/MS neg |
| eicosapentaenoate | Lipid | Essential fatty acid | LC/MS neg |
| docosahexaenoate | Lipid | Essential fatty acid | LC/MS neg |
| dihomolinolenate |  |  |  |
| arachidonate | Lipid | Long chain fatty acid | LC/MS neg |
| adrenate | Lipid | Long chain fatty acid | LC/MS neg |
| carboxy4methyl5propyl2furanpropanoate | Lipid | Fatty acid, dicarboxylate | LC/MS neg |
| propionylcarnitine | Lipid | Fatty acid metabolism (also BCAA metabolism) | LC/MS pos |
| deoxycarnitine | Lipid | Carnitine metabolism | LC/MS pos |
| carnitine | Lipid | Carnitine metabolism | LC/MS pos |
| HETE | Lipid | Fatty acid, monohydroxy | LC/MS neg |
| myoinositol | Lipid | Lysolipid |  |
| glycerophosphorylcholine | Lipid | Glycerolipid metabolism | LC/MS pos |
| Palmitoleoylglycerophosphocholine | Lipid | Lysolipid | LC/MS pos |
| oleoylglycerophosphocholine | Lipid | Lysolipid | LC/MS pos |
| Palmitoylplasmenylethanolamine | Lipid | Lysolipid | LC/MS pos |
| Palmitoylglycerophosphoinositol | Lipid | Lysolipid | LC/MS neg |
| Arachidonoylglycerophosphoinositol | Lipid | Lysolipid | LC/MS neg |
| glycerol | Lipid | Glycerolipid metabolism | GC/MS |
| glycerol3phosphate | Lipid | Glycerolipid metabolism | GC/MS |
| cholesterol | Lipid | Sterol/Steroid | GC/MS |
| pregnendioldisulfate | Lipid | Sterol/Steroid | LC/MS neg |
| Cortisol | Lipid | Sterol/Steroid | LC/MS pos |
| androsten3beta17betadioldisulfate1 | Lipid | Sterol/Steroid | LC/MS neg |
| glycocholenate sulfate | Lipid | Bile acid metabolism | LC/MS neg |
| xanthine | Nucleotide | Purine metabolism, (hypo)xanthine/inosine containing | LC/MS pos |
| Urate | Nucleotide | Purine metabolism, urate metabolism | LC/MS neg |
| Uridine | Nucleotide | Pyrimidine metabolism, uracil containing | LC/MS neg |
| pseudouridine | Nucleotide | Pyrimidine metabolism, uracil containing | LC/MS pos |
| catecholsulfate | Xenobiotics | Benzoate metabolism | LC/MS neg |
| ippurate | Xenobiotics | Benzoate metabolism | LC/MS neg |
